# Pediatric Cardiac Surgery in Low-and Middle-Income Countries: Present Status and Need for a Paradigm Shift

**DOI:** 10.3389/fped.2019.00214

**Published:** 2019-06-13

**Authors:** John S. K. Murala, Tom R. Karl, A. Thomas Pezzella

**Affiliations:** ^1^Cardiothoracic Surgery, University of Texas Southwestern Medical Center, Dallas, TX, United States; ^2^Professor Emeritus Johns Hopkins University, Baltimore, MD, United States; ^3^International Children's Heart Fund, Worcester, MA, United States

**Keywords:** humanitarian medicine, cardiac disease, developing countries, children, congenital, rheumatic heart disease

## Abstract

In low and mid-income countries, there has been a 50% global decrease in the incidence of preventable deaths of children since 1990. However, the mortality from non-communicable diseases (NCD) such as congenital heart disease (CHD) has not changed. Of the estimated 1.3 million children born with CHD annually, over 90% do not have access to cardiac care. With the increasing fertility rates in sub-Saharan Africa, the health burden of CHD will increase as well. Over the last 30 years much has been achieved with short term cardiac medical missions. However, much remains to be done to provide long term solutions needed to achieve the sustainable development goal of reducing deaths of children <5 years of age. This review discusses the present status and the need for a paradigm shift to achieve long term sustainability.

## Introduction

**Figure 1:**
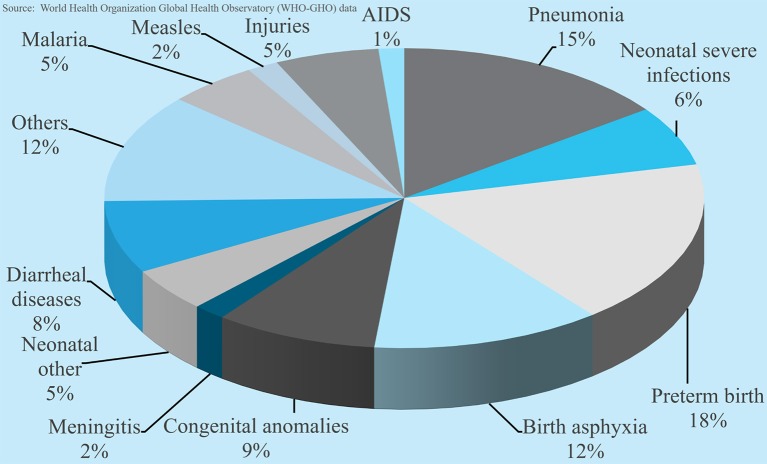
Causes of global mortality for children <5 years' age.

The global population is approaching 8 billion. Over the last few decades progress has been made in reducing maternal and child mortality as well as diseases such as malaria, tuberculosis, and HIV. The UN, WHO, and UNICEF data show that global mortality in the first 5 years of life declined from 93 deaths per 1,000 live births in 1990 to 39 in 2017 (58%) (1, 2). The decline was over 50% in 144 of 199 countries and 1/3 of those countries reduced their mortality rates by 67%. However, the estimated under-5 mortality for sub-Saharan Africa is 76/1,000 live births. Six countries from this region have mortality rates > 100/1,000 live births, among the highest in the world. This translates to 6 million children per year (or 16,000 per day) dying before their 5th birthday (3). Figure 1 shows the causes of death in this age group.

The United Nations Development Program (UNDP) sustainable development goal for 2030 is to reduce under-5 mortality to 25 per 1,000 live births. We know from the current UNDP data that roughly 70 million children may die before reaching their 5th birthday and most will be from sub-Saharan Africa (2). Nine out of 10 children living in extreme poverty ($1.90/day family income) will be from this region. Importantly, the countries with the lowest income and highest fertility rates are from sub-Saharan Africa. There are multiple reasons for poor healthcare access among children, including lack of political commitment, maldistribution of resources (especially financial support), human resources, and lack of collective will. The time to act is now if we are to reach our sustainable goal by 2030 (2).

**Figure 2:**
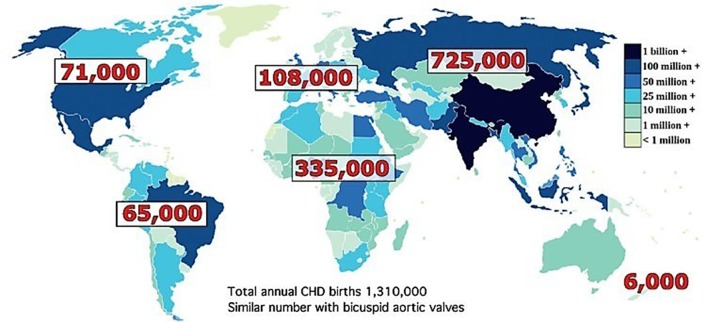
Total annual birth of children with CHD by continent wise. Total number is roughly 1,310,000. Similar number with bicuspid aortic valves. Reproduced with permission from Hoffman (4).

The world has often focused on communicable diseases as they are public health issues. In the last three decades there has been a fall in number of new HIV infections by 30% and over 6.2 million lives saved from malaria (2). However, the non-communicable diseases (NCD) such as cardiovascular diseases (CVD) have been gradually reaching epidemic proportions, causing an increased health care burden. According to WHO data, out of the 17 million premature deaths (under the age of 70) due to NCD in 2015, 82% are in low and middle income countries (LMICs), and 37% are caused by CVDs. Recent estimates for the incidence of congenital heart disease (CHD) are in the vicinity of 8–12/1,000 live births (4). In addition to CHD, the prevalence of acquired heart disease among children e.g., rheumatic heart disease (RHD) is also high in Africa and Asia. The approximate incidence of RHD in the African population ranges from 2.7 to 20/1,000 population (5). In Africa alone, of the 50 million live births annually, at least 335,000 will have CHD (Figure 2) and many more will develop RHD. Less than 5% have access to cardiac care. Without availability or accessibility to cardiac care one in three children born with CHD die within the first month of birth (4). It is estimated that 1.3 million children are born each year in the world with CHD (Figure 2). However, <100,000 have access to heart care leaving over one million each year without care. The cumulative numbers create a sizable back log. There is very little data about the prevalence of CHD or RHD from LMICs. The reasons are multifactorial. In addition to mortality from heart disease many of these children also die from infectious causes prevalent in these countries and since prevalence is determined by both incidence and survival it becomes harder to predict (6).

To achieve the sustainable development goal of 2030, care of CHD needs to be an integral part of the big picture.

## Common Goal

The common goal should be a universal reach of cardiac care with a concurrent decrease in mortality and morbidity from CHD. Presently there is a wide disparity in cardiac facilities. Over 70% of the facilities reach <20% of the world's population, leaving over 90% of children born with CHD without any access to cardiac care. In a detailed review, Bode-Thomas and Olga et al. have outlined all the challenges in the management of CHD in developing countries, and possible solutions (6, 7). In our review our aim is to discuss the practical aspects of dealing with the neglected cohort of CHD patients, review current efforts, and discuss possible future plans. We believe in the need for a paradigm shift i.e., a change in our thinking and strategies if we are to achieve the goals for 2030.

## Current Programs

**Figure 3:**
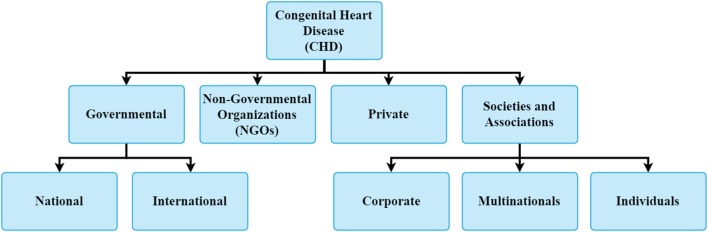
The various organizations providing cardiac care in LMICs.

The current organizations involved in the care of children with CHD are shown in Figure 3. Congenital cardiac surgery programs exist as part of larger adult cardiac surgery programs or as separate divisions. They are parts of general hospitals, separate children's hospitals or stand-alone pediatric cardiac centers. The various cardiac programs can be categorized as follows:

1. Developed (established) programs
2. Developing programs
3. “*De novo*” programs
4. Restarting programs (failed or abandoned)

There are two types of volunteer cardiac surgery programs described by Dearani et al. (8, 9). They include:

1. Short term (1–2 weeks or once or twice per year) medical missions. Some of the missions in the world are listed in Table 1. For a more comprehensive list please refer to Nguyen et al. (10).
2. Long term, pairing programs, or imbedding models are programs in the developed world partnering with programs in the developing world for long-term partnerships, commonly 5–10 years. The focus is on education, training, skill set development, improving outcomes, quality control, and long-term sustainability.

**Table 1:** Some groups doing cardiac surgical missions.

**Number**	**Name of organization**	**Websites**
1	American College of Surgeons	https://www.facs.org/ogb
2	Bambini/Cardiopatici Nel Mondo	www.bambinicardiopatici.it/
3	Be Like Brit	https://www.belikebrit.org/
4	Cardiostart	https://cardiostart.org/
5	Chain of hope	https://www.chainofhope.org/
6	Children's Heart link	https://childrensheartlink.org/
7	Crudem	http://crudem.org/
8	CTSNET	https://www.ctsnet.org/
9	European Association for Cardiothoracic Surgery	https://www.eacts.org/
10	Earth Med	https://www.earthmed.org/
11	European heart for Children	http://www.europeanheartforchildren.com/
12	For hearts and Souls	http://forheartsandsouls.org/
13	Foundation Mauritanienne duCoeur	www.mauritanie-coeur.org
14	Frontier Lifeline	http://www.frontierlifeline.com/
15	Gift of Life International, Inc.	http://www.giftoflifeinternational.org/
16	Global Healing	https://globalhealing.org/
17	Global Heart Network	https://globalheartnetwork.wordpress.com/
18	Global Impact	https://www.charity.org/index.html
19	Haitian Hearts	http://www.haitianhearts.org/
20	Healing the Children	http://healingthechildren.org/
21	Hearts Around the World	http://www.heartsaroundtheworld.org/
22	Heart to Heart	https://www.heart-2-heart.org/
23	Heartbeat International Foundation	https://www.heartbeatsaveslives.org/
24	Heart Care International	https://www.heartcareintl.org/
25	Hearts for All	https://www.coeurspourtous.ch/
26	International Aid	http://www.internationalaid.org/
27	International Children's Heart Foundation	https://www.babyheart.org/
28	International Children's Heart Fund	http://www.ichfund.org/
29	Heal A Child	https://www.heal-a-child.org/
30	Magdi Yacoub Foundation	https://myf-egypt.org
31	MAP International	https://www.map.org/
32	Mending Kids International	https://www.mendingkids.org/
33	Mercy Ships	https://www.mercyships.org/
34	Novick Cardiac Alliance	https://cardiac-alliance.org/
35	Open Heart International	https://ohi.org.au/
36	Palestine Children's Relief Fund	https://www.pcrf.net/
37	Pan-African Academy of Christian Surgeons	https://www.paacs.net/
38	Physicians for Peace	http://www.physiciansforpeace.org/
39	Project Kids	https://www.projectkidsworldwide.org
40	Project Haiti Heart	http://projecthaitiheart.org/
41	Project Hope	https://www.projecthope.org/
42	Project Open Hearts	http://www.poh.org/
43	Project Medishare	http://projectmedishare.org/
44	Russian Gift of Life	http://rgolusa.org/
45	Samaritan's Purse-International Relief	https://www.samaritanspurse.org/
46	Save A Childs Heart Foundation	https://www.saveachildsheart.org/
47	Surgeons of Hope Foundation	https://surgeonsofhope.org/
48	Team Heart- Rwanda	https://teamheart.org/
49	The Heart of a Child Foundation	http://www.heartofachild.org/
50	The Childrens Lifeline	http://childrens-lifeline.org/
51	Vina Capital Foundation	https://vinacapitalfoundation.org/en/
52	Walter Sisulu Pediatric Cardiac Foundation	https://wspcf.wordpress.com/
53	World Heart Federation	https://www.world-heart-federation.org/
54	World Pediatric Project	https://www.worldpediatricproject.org/

*Source- International Children's Heart Fund website (http://www.ichfund.org/Content/Organizations.htm). For more comprehensive list please refer to Nguyen et al. (10)*.

## Short Term Medical Missions

Short term cardiac medical missions have been offered for many years in Asia, Africa, Central and South America. In the earlier era these missions only performed closed heart procedures before gradually introducing open heart surgery. In later stages some of the native surgeons and cardiologists were sent to developed countries for training. These countries receiving short-term missions usually fell into three categories including:

1. Developing countries with *in-situ* cardiac programs.
2. Developing countries which will likely never have a program of their own (e.g., Caribbean and surrounding islands with a population of 40 million and the Pacific with similar populations).
3. Previous failed programs

Over the last two decades there has been a proliferation of short term missions, especially in Asia and Africa. Based on different strategies, some of these missions have been improperly labeled as “medical/surgical safaris” (11, 12). The authors believe that the criticism may be unfair as these “medical/surgical safaris,” though not perfect, were able to help scores of patients who otherwise had no access to surgery. Good hearted and well-meaning surgeons, cardiologists, nurses, and other professionals often take vacation time to travel to impoverished areas, putting their personal safety aside. However, it is difficult to discern how many of the host units have become self-reliant. This was highlighted in a study of 26 medical missions in sub-Saharan Africa (13). The authors concluded that the current model of collaboration via short term medical missions appears sub optimal for skill transfer and suggested deeper involvement of universities, governmental institutions, and visiting teams. The communication, networking, defined training, and long term goals need to be defined to achieve the complex goal of a sustainable program. We believe that there is a role for these short-term missions. In a detailed study of the financial implications of their many short term missions to LMICs, Cardarelli et al. have shown the cost effectiveness of intervention and the benefit to the society (14). In 2015, 446 patients received intervention in 10 LMICs at a total cost of $3,210,873. Each intervention was estimated at $171 per disability adjusted life-year averted. Each survivor potentially gained $159,533 in gross national income per capita during his or her extended lifetime (14). It is difficult to assess the number children who have come to developing countries over the years for free surgery. However, these children become productive members of society on a long term basis.

Here we discuss the present condition of the medical missions and possible future strategies. There are many pertinent questions. Do we continue with these short term teams? How do we better utilize human resources? How can we build programs in low resource countries? Can there be a better coordination between non-governmental organizations (NGOs)? What is the exit strategy? Can there be a unified approach? What are the long term strategies? What are the possible strategies to maximize the benefit? The big questions are sustainability, accountability, transparency, and training. There is no “one size fits all” strategy.

## What we Know Thus Far

**Figure 4:**
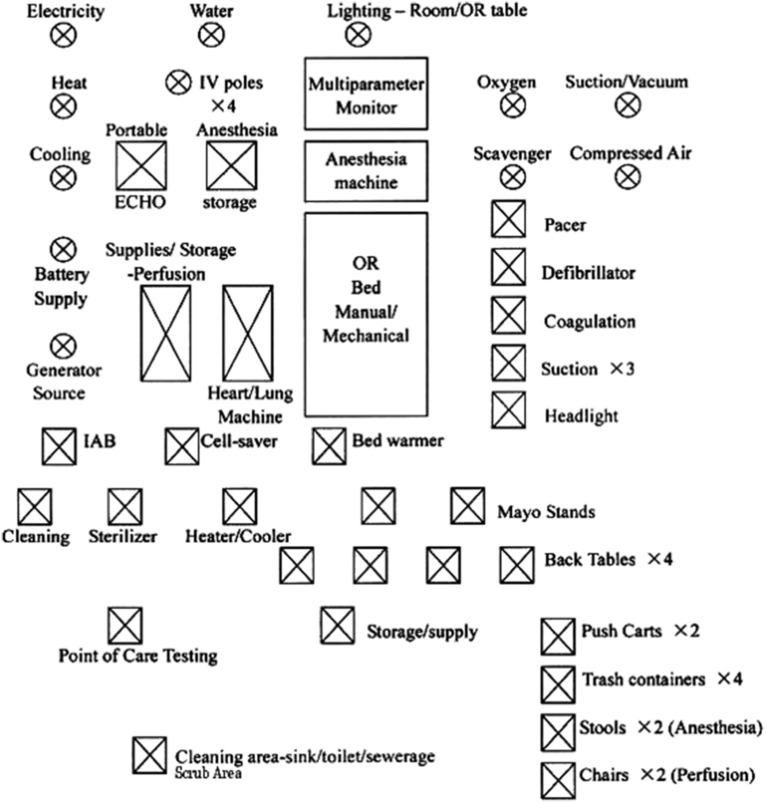
Essentials for a cardiac OR (OR, Operation rom; IV, Intravenous pole; IAB, Intraaortic Balloon pump). Reproduced with permission from Dr.Pezzella http://www.ichfund.org/Content/OR-ICU_lists.htm.

**Figure 5:**
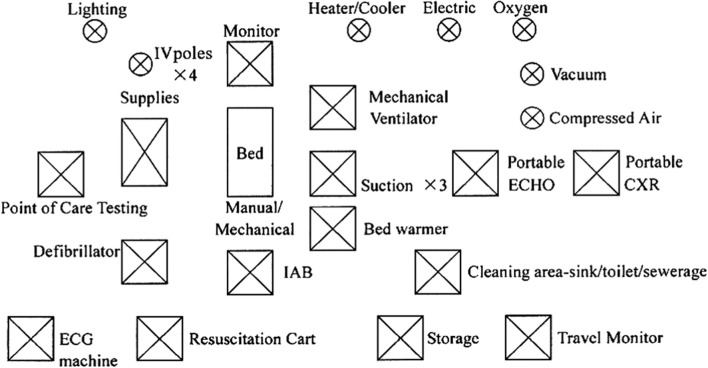
Essentials for an ICU (IV, Intraveonus; IAB, Intraaortic Ballon pump). Reproduced with permission from Dr.Pezzella http://www.ichfund.org/Content/OR-ICU_lists.htm.

1. PERSISTENCE AND CONTINUITY: In one of the largest experiences in starting multiple pediatric cardiac centers in Russia, Young et al. acknowledged the importance of careful site selection based on demographic research as well as initial and secondary site assessments (15). Surgical education in the form of donor continuity and annual surgical education missions are important to achieve pairing of two cardiac centers, one of which is an established program. As the programs evolve and mature regular evaluations help with growth and sharing (“cross fertilization”).
  This was further reiterated by Dearani et al. in their review of humanitarian efforts in developing countries and emerging economies (9). System factors influencing the delivery of healthcare include accessibility, availability, awareness, and affordability. They identified several key areas needing attention for a successful medical mission. They include: (1) Background study of the host country, (2) Identifying a dedicated host team with definable leader, (3) A Memorandum of Understanding (MOU) which stresses the exit plan, (4) Government help must be included, (5) Consider reliable NGOs for low cost items as well as locally available disposables and devices, (6) Successful programs depend on vision, appropriate skill sets, accessibility, availability, awareness, affordability, and action plan. They conclude that humanitarian, medical, and surgical outreach activities should focus on education and sustainability reserving “surgical tourism” for those countries that will likely never have the capability to have free standing cardiothoracic programs (9).
2. COOPERATION OF NGOs: Multiple organizations working in synergy to realize a common goal is crucial to success. Frigiola et al. highlight the success of the Bambini Cardiopatici Nel Mondo association and their cooperation with various NGOs, which has paved the way for various cardiac programs in Africa and beyond (16). Similarly, Dearani et al. have also described the importance of developing partnerships between governments and communities (8). The factors essential for successful partnerships include shared responsibility, pooling of resources, open communication, quality control, proper channeling of resources, and auditing (16).
3. FOCUS ON CHD: It is common knowledge that the priority in developing world is to combat communicable diseases. Very little attention is given to congenital or acquired heart disease in children. We must recognize the problem and the contributing factors, provide access to cardiac operations for common congenital diseases, and provide infrastructure through partnerships with governments and NGOs. When a program is initiated, starting with adult cardiothoracic surgery before pediatric cardiac surgery may be logical. Other important points include increasing human resources in health care via training programs and ongoing research with quality improvement (17).
4. ON SITE ISSUES: Once an “onsite” campus is identified, there are many factors which determine the success of the mission. The donor (NGO) and the host (on site) need to better coordinate the necessary needs and wants. Molloy et al. have identified the many on site issues and their possible solutions (18). The issues related to infrastructure, biomedical equipment, disposables (including devices and drugs), patient selection, human resources, training, quality control, security, credentialing, malpractice, finances, and host issues have all been well described. The key is to prepare oneself for all possibilities. Dr. Graham Nunn, a retired congenital heart surgeon from Australia, has spent the last 20 years of his career traveling to Papua New Guinea on annual short term missions. On request from the authors, he has sent the following communications about his experience and insight, which are summarized in Tables 2, 3. Most importantly, he describes the need to have back up for failures of blood gas analyzers, autoclave, heart lung machines, and ventilators. In Figures 4, 5, we detail the essentials needed for a cardiac operating room (OR) and intensive care unit (ICU), respectively.
5. VIEW FROM HOST PROGRAMS: Africa has many unique problems. They are over 1 billion in population with more than 50% under 25 years of age (5, 6, 19). Challenges include political instability, civil unrest, refugee populations, apathy, maldistribution, corruption, mounting debt, and frequent conflicts. The communicable diseases dominate the health programs. Only a small portion of gross domestic spending is for health care. The burden of CHD is only a part of the problem with RHD remaining the most common cardiac disease. Treatment often requires the availability of both adult and pediatric cardiac surgeons. However, surgeons who visit on medical missions from western countries are not necessarily trained to operate on and treat RHD. Furthermore, after valve replacement, long-term anticoagulation remains a problem in remote areas of Africa. Surgical training with hands-on approaches is another difficulty (5, 19).
6. FINANCES: This is the biggest predicament of the short-term mission trip. In a recent study published by Dr. William Novick and team, the humanitarian pediatric cardiac surgery programs to LMICs showed that they are very cost effective on a long-term basis (14). Cost cutting is achievable with local corporations and distributors.

**Table 2:** The list of resources and the contingency plans for a short-term mission (Dr. Nunn).

**Resources**	**Have contingency plans for**
We should take adequate staff. Limit doctors and take more nurses, OR, ICU, anesthetic, and floor staff	Loss of water, Oxygen, and Electrical supply to OR and ICU. It will happen at some point
Biomedical staff are important ICU staff must back up local staff	Hand ventilating every patient in OR and ICU
Team manager role is critical	Emergency evacuation
We should select people who are “lateral thinkers” and who are willing to innovate in given circumstances- especially surgical and perfusion teams	Dealing with all possible post-operative complications in ICU
We should take enough materials and equipment	Local equipment failure e.g., we should have portable monitors in case of failure of standard monitors
We must take enough drugs for all contingencies	Emergency and resuscitation drugs
We must be prepared for inadequate blood banking support. We must take hemostatic agents e.g., Tranexamic acid and if possible, Factor VII A components	Provision for using fresh whole blood if components are not available. Will need to stock with blood drawing kits
We must take enough instruments/drapes/dressings	Local sterilization equipment failures

**Table 3:** The Do's and Don'ts in a short-term mission (Dr. Nunn).

**DOs**	**DON'Ts**
The trip should only happen at the invitation of the host country	We should not force a team onto the host if they are not ready for us
We should engage with the local administrators and provide positive feedback each trip and ask them what they would like to achieve on the next trip and try to put that into practice	We should not impose our strategy on the host. Successful teams are those whose mission aligns with that of the host
Training must be hands on and very much “do as I do”, rather than “do as I say”	We should not compromise on patient safety
We must work within the local politics, local trainees …. Competencies……	We just do not know all the background linkages between people in another society and can quickly offend
These things take time and certainly the worst thing is to try to tell the local administrators what to do	This also applies to local funding We have to say to ourselves, “this is the reality, how are we going to get done what we came here to do?” More importantly though, local funding needs to build and sustain the program that develops from these visits, so it is the essential ingredient for home grown success long term
Well one thing we will do is do it ethically and without compromise and try to live by example	We must be wary of using the voluntary work as a conduit for private practice
We should try to achieve outcomes that are the same as our parent institution	Deaths will be long remembered and will not lead to good will amongst the administrators and providers of funds when we are not there
We should select patients who can expect a good outcome and can reasonably be expected to be helped by the local team when they get up to speed	This means that heroic surgery should not be done. Just because the patient will die if we do not “have a go” is the worst way to select the patients for surgery
Attend socials but limit them so we can rest and concentrate on work ahead	Try not to spend every evening going to social functions. It is natural for a team to want to socialize but those working days are hard and no one can perform to their own standard with that dragging them back each day. We do not do it at home so how can we think we are super human on one of the trips
We should take a very long-term view about how quickly the local team will come up to speed	We must stop being critical of the hosts
Security is very important and must be provided by the local teams. Professional indemnity must be granted from the government of the country	In unsafe areas we should not venture on our own- no “Bravado” actions
Immunization must be a pre-requisite for all team members	

## A Model for Establishing a Long-Term Program

There is a collective experience of over 30 years dealing with short term missions. There are many lessons learned and few can be used to model a long term program. We have many years of catching up to do. The global challenge of CHD is ever increasing. In order to achieve the shared goal of accessible cardiac care for every child, the objective should be more global collaboration and shared strategies. This strategy depends on several questions. There is an established program which needs further help? Was there a program which is now closed and needs rebuilding? If there are no existing programs, does the country need or want one?

The task of providing cardiac care and surgery for all children in need appears daunting. There is a global shortage of skilled workers in congenital cardiac care. Leblanc proposes a “KISS” (keep it simple and safe) approach (20). Everything needs to be organized and simplified. This planning includes: site evaluation, training, on site infrastructure development, necessary OR and ICU equipment and training, laboratory training, blood banking, and administrative work. Despite hard work and tremendous effort, experience shows that it takes many years to achieve sustainable growth. Corno, in his review, writes that a successful long-term program must have the following requirements: high quality measurable outcomes, sustainability, scalability, and efficacy (12).

Here we review some suggested ways to achieve a successful long-term program:

1. SHARED VISION: If our common goal is to provide cardiac care for all children in the world, the existing societies (Society of Thoracic surgery (STS), American association of Thoracic surgery (AATS), Asian Society of Cardiovascular and Thoracic Surgery (ASCVTS), European association of Cardiothoracic Surgery (EACTS), World Society of Pediatric and Congenital heart surgery (WSPCHS), World congress of Pediatric cardiology and cardiac surgery etc.) must, as well as the CTS Net play an important and expanded role in a top down approach. In addition to establishing volunteer platforms and conducting annual sessions of development of cardiac surgery in LMICs, there should to be a larger cooperation and collaboration between various societies. One such example is the Cape Town declaration on access to cardiac surgery (especially the scourge of RHD) in the developing world (21). This top down approach creates awareness and fosters collaboration between international cardiac surgery societies, industries, and government. Their aim is to establish international working groups from the above groups to evaluate and endorse development of cardiac care in LMICs and training of surgeons and other professionals in these countries (21). Similarly, the “global statement” sponsored by the Pediatric Cardiac Intensive Care Society is another step for collaboration and top down approach. The statement fosters partnerships, education, and training (22).
2. “SPARK PLUG”: An essential component for a long term success is a “spark plug.” The term was used by Dr. Terry Davis, a congenital surgeon, from Ohio, in a private conversation. He described it as an “organization or individual, often from the host country, who is a dedicated leader, focused, invested, and physically present in a local program on a long term basis.” There are many examples of “spark plugs” in the world. We have previously described work of some organizations in Russia (15) and Africa (16). One such is Dr. Efrain Montesinos. He took an early retirement from the United States and went back to his native country of Peru. He worked to establish a cardiac surgery program among the indigent (23). He and his wife were physically present in Peru, navigated the bureaucracy and the deficiencies of the system, and started short-term medical missions in an old prison ward in the late 1990s with the support of philanthropic societies and hospitals in US. This initiative over the years has resulted in one of the major cardiac surgery departments in Peru. He did not live to see the entire success as he died in 2007, but his legacy remains (23). Another “spark plug” is Dr. Aldo Castaneda. His hard work, persistence, and dedication led to the establishment of a sustainable pediatric cardiac surgical program in Guatemala City, Guatemala (11). Similarly, other surgeons such as Alain Carpentier, Magdi Yacoub, Alain DeLoche and many others have all been “spark plugs.”
3. COMMUNICATION AND COORDINATION: We believe there are enough resources for many LMICs if there is better coordination between the various donor and host organizations (NGOs, corporates, individuals, governments, and hospitals). The inadequate communication between these organizations leads to insufficiency and duplication of work. Centers of excellence in the developed countries need to be identified. Coordination between these centers, the NGOs, and the host countries can be very beneficial. Within the host country there is usually a vast maldistribution of medical care. The location of the hospital should be selected based on population distribution, urban/rural location, income divide among the people, availability of materials, and ease of access of the facility. A formal MOU between NGOs/charitable trusts, societies, governments, corporations, and individuals is encouraged. Developing regional “hubs” which serve smaller satellite centers for a geographical zone or region would be ideal. Examples of “hubs” could include: (1) Europe looking after certain African countries, (2) North America helping Central and South American as well as the Caribbean countries, (3) Australia helping with the Pacific Islands and some Asian countries, (4) Israel and Saudi Arabia helping with the surrounding countries, and (5) Emerging economies such as China India, and South Korea also helping with training and material support. This method is more logistical and feasible as these countries are often in the same time zones. The question would be who would oversee such an organization. What is really needed is a worldwide body to divert allocation- but we all know this is unlikely. Some of these initiatives were detailed by Dr. Cox in his presidential address in 2001 (24). The “hub” philosophy may be ideal, but this should not preclude people from going anywhere/everywhere to help. This goes with the philosophy “perfection may be an enemy to good.”
  One of the earliest successful partnerships which resulted in a long-term program is the Vien Tim institute in Vietnam. It was a result of partnerships with government, Carpentier foundation, and numerous other charities. Over the years the institution grew and currently performs over 1,500 open heart operations annually (25, 26). Another successful partnership is the cooperation between the model of IRCCS Policlinico San Donato and the Bambini Cardiopatici nel Mondo association for CHDs. Many countries in Northern Africa, Middle East, Eastern Europe, and Latin America have benefitted from their collaboration. Cardiac surgery departments evolved in countries such as Cameroon, Syria, Northern Iraq (Kurdistan), Peru, and Romania (16). The Shisong Cardiac Center Project in Cameroon is another great example of cooperation between various organizations, Bambini Cardiopatici nel Mondo Association and Hospital (IRCSS Policlinico-San Donato) another Italian NGO (Cuore Fratello) and local faith-based hospital (Tertiary Sisters of Saint Francis). They started work to establish cardiac care in 2002 and in 2009 were successful in establishing an autonomous institution which is the largest cardiac care center in Cameroon (27). Another good example of “taking cardiac surgery to the people” is the model from Egypt which is mainly funded by donations from Egyptian people (28).
  Some practical aspects of coordination include:
  - Choosing one country of interest and then coordinating with all agencies interested in that country. This is idealistic and may be difficult to follow. But an attempt can be made. Young et al. have described the establishment of 6 new congenital programs over 25 years of focused approach in Russia (15).
  - Teams going sequentially for a defined period creating an overlap of resources. This is hard to achieve with little coordination between the involved NGO's. This has been the request from many of the host countries from Africa (19).
4. TRAINING: The strategy for training is very important. Training which involves administrative, clinical, and research areas should be an effective component of a mission. However, the most difficult piece is the hands-on training in the OR. The training can be divided into several phases (1) observational training in a developed center (2) hands-on training by visiting teams on site, and finally (3) having an embedded on-site mentor, consultant, or proctor. The role of retired or semi-retired surgeons is invaluable for a fixed 1 month stay or longer (27). The role of senior surgeons is very valuable as shown by Dr. Aldo Castaneda in the Guatemala experience (11). There is a role for cross training (e.g., OR nurses can be trained for perioperative care of patients, the perfusionists can be trained to help in closure of wounds, etc.). This is a cost-effective strategy for a starting program. This is like some sort of “military special forces” who are trained as “multipurpose workers.” This may be better in the initial stages for building a team philosophy. Previously, Australia, the United Kingdom (UK), and the US have trained many surgeons from developing countries helping them establish programs in their own countries. However, the current immigration and training regulations preclude easy access.
  Recently, countries such as India have become the new hub for training as they have increased the number of cardiac programs. There are surgeons being sent there for hands on training (29). Bastero et al. have presented 4 models of partnership models in pediatric cardiac surgery and cardiac intensive care programs in LMICs (26). The sustainable model of pediatric cardiac surgery program in Mexico which is a private-public partnership, with help from NGO and the Texas Heart Institute in the US is one example. Similarly, sustainable programs have been developed in India and Vietnam with education and training of the nurses and medical staff via organizations in the UK, France, and Australia. Similarly, Chain of Hope helped in developing a program in Jamaica. These examples prove the importance of partnerships (26).
  World societies such as WSPCHS, EACTS, and the hubs can help with periodic education programs. Simulation training is invaluable. Other modalities like use of 3-D technology, virtual reality, and augmented reality may also play a role in the future. Remote training can be achieved with telemedicine. It cannot be stressed enough how invaluable this technology is for remote discussions, training, and consultations. However, this can never replace hands-on training for the local teams.
5. MATERIAL SUPPORT: Developing infrastructure is a challenge. There should be development in all supporting departments such as the OR, ICU, anesthesia, perfusion, biomedical, cardiology, pharmacy, nursing, blood bank, and administrative support. Basic infrastructure needs to be provided. The equipment/disposables, devices, sutures, instruments and drugs are expensive. Here the donations of equipment (ideally <10 years old) in good condition from developed centers may be invaluable. There are aid agencies which refurbish used equipment including perfusion machines which can be useful. The disposables and materials may be cheaper to buy locally. Post-operative follow-up is also crucial to success of teams. A continual supply of drugs needs to be made available. A final and important question is where will the financial resources come from (e.g., government, private institution, charitable trust, or out of pocket)?
6. EXIT STRATEGY: Long term programs may be more successful if there are already “*in-situ*” cardiac programs. However, there needs to be an exit strategy to prevent the creation of an entitled program or dependent program with no growth for the local teams. One successful venture by project HOPE is the pediatric cardiac surgery program in Shanghai, China spearheaded by Richard Jonas. The *in-situ* program which was performing 200 cases annually in the past is now performing over 4,000 operations annually on its own (30). This is a successful cooperation among various players in the country of interest but there was a clear exit strategy and the host hospital became self-reliant.
7. DATA: For long term success a data base needs to be maintained for evaluation of progress. The epidemiological studies pertaining to incidence and prevalence in CHD and RHD need to be better understood. Then the center specific/surgeon specific data can be evaluated. This includes the cases performed and their follow up. There could be a better role for the WSPCHS in the US and the European Congenital Heart Surgeons Association in Europe to spearhead the data acquisition of these programs.

## What is the Future?

We have written about the past and what is being done presently by many organizations.

There is very little written about the reasons for failure of programs in developing and developed countries. It is human nature not to talk about failures. We have identified a few reasons for failure of projects. Often the reasons are due to (1) unsustainability and no exit strategy (e.g., Haiti and Nigeria), (2) “Bridge too far”-Caribbean and many Pacific islands are spread over hundreds of islands with small populations and it would be impractical to have individual cardiac centers in all islands (e.g., Cook Islands, Kiribati, Tonga, Fiji). Developing regional hubs for them is probably the right thing. The Dominican Republic acting as a hub for the Caribbean and either Australia, New Zealand, Hawaii or Papua New Guinea can be developed as a hub for the Pacific, (3) poor results-often the surgeon is blamed but it is multifactorial and a system failure, (4) financial issues are one of the most glaring problem. Many programs in Central and South America are finding it difficult to run their current cardiac programs (e.g., Guatemala), (5) personal egos of bureaucrats and medical professionals, (6) safety and war situation such as programs in Middle East, and finally (7) lack of government support.

We believe that much depends on developing regional hubs and using embedding as an effective tool. Dr. Aldo Castaneda once said “Development of a sustainable pediatric cardiac program in emerging countries presents many difficult challenges. Hard work, perseverance, adaptability, and tolerance are useful aptitudes to develop a viable program in an ‘emerging' country. We are not in favor of medical surgical safari efforts, unless these efforts include training of a local team and eventual unit independence. It helps if an experienced (± senior/retired) surgeon leads this effort on a full-time, pro-bono basis. Local and international fund raising is essential to complement vastly insufficient government subsidies” (11).

“Embedding” involves a trained surgeon, retired or on sabbatical spending long periods of time helping a center. It could be 6 months or 1 year. One of the authors (Pezzella) has had the experience of spending long periods of time in China, Vietnam, and other countries (31, 32). The “embedding” program could be sponsored by associations like AATS (Graham traveling scholarship), STS, EACTS, and ASCVTS. These will be cost effective in the long term. As the hands-on training is becoming harder for new surgeons the “embedding” program may be an answer for onsite training.

## Summary

Much has been written by scores of individuals and organizations about their experience in providing cardiac care for LMICs. Much has been done and lessons learned. We believe that a comprehensive global cooperation is urgently needed if we are to provide heart care to every child born and fulfill our goal of sustainable care by 2030. There needs to be active participation from different cardiac societies, collaborations with NGOs and other organizations. This needs to be on the top of agenda for their organizations. Regional hubs need to be identified and supported. Training needs to be coordinated and we hope that there is a fire lit in all cardiac surgeons/cardiologists to help with this cause. We hope for many “spark plugs” who are willing to give their time for training and help with any unit on a long-term basis. The time to act is now.

## Data Availability

All datasets for this study are included in the manuscript and/or the supplementary files.

## Author Contributions

JM has done over 50% of the draft. TK has introduced concepts and contributed to 20% of the draft. AP has initiated this review and helped with literature search and thus helped with 30% of the draft.

### Conflict of Interest Statement

The authors declare that the research was conducted in the absence of any commercial or financial relationships that could be construed as a potential conflict of interest.
